# ISway: a sensitive, valid and reliable measure of postural control

**DOI:** 10.1186/1743-0003-9-59

**Published:** 2012-08-22

**Authors:** Martina Mancini, Arash Salarian, Patricia Carlson-Kuhta, Cris Zampieri, Laurie King, Lorenzo Chiari, Fay B Horak

**Affiliations:** 1 Department of Neurology, School of Medicine, Oregon Health & Science University, 505 NW 185th Avenue, Beaverton, OR, 97006, USA; 2 Biomedical Engineering Unit, Department of Electronics, Computer Science & Systems, Alma Mater Studiorum-Universita’ di Bologna, Viale Risorgimento 2, 40136, Bologna, Italy; 3 Functional and Applied Biomechanics Laboratory, Rehabilitation Medicine Department, National Institutes of Health, Clinical Center, Building 10, MSC 1604, Bethesda, MD, 20892, USA

**Keywords:** Postural control, Accelerometers, Inertial sensors, Parkinson’s disease

## Abstract

**Background:**

Clinicians need a practical, objective test of postural control that is sensitive to mild neurological disease, shows experimental and clinical validity, and has good test-retest reliability. We developed an instrumented test of postural sway (ISway) using a body-worn accelerometer to offer an objective and practical measure of postural control.

**Methods:**

We conducted two separate studies with two groups of subjects. *Study I: sensitivity and experimental concurrent validity*. Thirteen subjects with early, untreated Parkinson’s disease (PD) and 12 age-matched control subjects (CTR) were tested in the laboratory, to compare sway from force-plate COP and inertial sensors. *Study II: test-retest reliability and clinical concurrent validity.* A different set of 17 early-to-moderate, treated PD (tested ON medication), and 17 age-matched CTR subjects were tested in the clinic to compare clinical balance tests with sway from inertial sensors. For reliability, the sensor was removed, subjects rested for 30 min, and the protocol was repeated. Thirteen sway measures (7 time-domain, 5 frequency-domain measures, and JERK) were computed from the 2D time series acceleration (ACC) data to determine the best metrics for a clinical balance test.

**Results:**

Both center of pressure (COP) and ACC measures differentiated sway between CTR and untreated PD. JERK and time-domain measures showed the best test-retest reliability (JERK ICC was 0.86 in PD and 0.87 in CTR; time-domain measures ICC ranged from 0.55 to 0.84 in PD and from 0.60 to 0.89 in CTR). JERK, all but one time-domain measure, and one frequency measure were significantly correlated with the clinical postural stability score (r ranged from 0.50 to 0.63, 0.01 < p < 0.05).

**Conclusions:**

Based on these results, we recommend a subset of the most sensitive, reliable, and valid ISway measures to characterize posture control in PD: 1) JERK, 2) RMS amplitude and mean velocity from the time-domain measures, and 3) centroidal frequency as the best frequency measure, as valid and reliable measures of balance control from ISway.

## Background

Postural control is the foundation of our ability to stand and to walk independently. Deterioration in postural control due to normal ageing or neurodegenerative disease such as Parkinson’s disease (PD) is associated with an increase in risk of falls incurred during activities of daily life [1,2]. Deterioration in balance control predisposes 68% of people with PD to fall at least once each year [3]. Although it is well-known that subjects with PD show postural instability in advanced stages of the disease [4-7], very few studies have investigated whether it is possible to identify abnormal balance in early stages of the disease, prior to starting antiparkinsonian medication [8-10]. Early identification of abnormal balance in patients with PD is important because new neuroprotective medications are currently being tested to slow the progression of PD. Neuroprotection needs to begin early in the disease, prior to significant loss of neurons. Also, early in the disease, it is often difficult to distinguish idiopathic PD from other, Parkinson-plus syndromes that have very different Prognoses and it is possible that sway characteristics will differ among these different basal ganglia diseases.

Currently, the most common way to evaluate postural control in the clinic is to use clinical rating scales that are limited by clinicians bias, insensitivity to mild impairments (ceiling effects), and poor reliability [11-13]. These limitations are serious concerns for clinicians and researchers who want to monitor disease progression, determine intervention efficacy or treat people with mild balance deficits [14]. Technology currently available for clinicians and researchers to measure postural control generally uses force plate analysis of center of pressure (COP) displacement during quiet stance [6,15]. Experimental studies have demonstrated the sensitivity of COP measures to postural disorders such as Parkinson’s disease [8,9] and to fall risk in the elderly [16,17]. However, force-plate-based posturography (or stabilometry) is quite large and expensive and requires a proper installation that may not be practical for clinical use.

Body-worn accelerometers (ACC) have been proposed as a portable, low-cost alternative to a force plate for measurements of postural sway [2,18-21]. Recently, we showed that ACC-based measures of postural sway are sensitive to balance disorders in patients with untreated PD [10]. Moreover, Najafi et al., demonstrated that ACC-based derived measures of center of mass during postural sway detect deterioration in patients with diabetic neuropathy [2]. These results suggest that such measures may provide a sensitive means of measuring subtle balance deficits in clinical settings.

Despite the potential advantages of accelerometric systems in clinical practice, they still have several drawbacks, such as the need to pre-process data and the question of how to translate sway measures into clinically understandable outcomes. However, the major limitation is that there is no consensus as to which sway-related measures should be considered. Studies have shown that root mean square (RMS) of the acceleration signal can be sensitive to test conditions (eye closure, standing on one foot), to ageing, and to history of falls [22-24]. We recently presented a relatively new measure of sway, “JERK”, as the most discriminative measure to differentiate sway in patients with untreated PD compared to age-matched control subjects [10].

In order to make ACC-based measures useful for clinical applications, it is important to assess their validity, sensitivity, and reliability, compared to gold standard lab and clinical assessments [25,26]. The relationship between the same postural sway measures calculated from the force plate COP and from ACC that would support the experimental validity of ACC measures have only been reported in three studies, the first by Adlerton et al. [20] in one-legged stance trials in healthy adults, the second by Najafi et al. [2] in diabetic patients, and the third by Whitney et al., [18] in healthy control subjects during different sensory conditions. No studies have shown experimental validity of ACC-based measures and force plate-based measures of postural sway for subjects with very mild balance deficits, such as those with untreated PD. Our previous study on ACC measures of postural control only showed which are the best measures that discriminate between untreated PD and age-matched control [10]. Moreover, to our knowledge, only one study has presented test-retest reliability of balance measures calculated from an ACC placed on the belt of young, healthy adults [27]. There are no studies that have evaluated test-retest reliability of postural sway in patients with PD and elderly subjects, or studies that have systematically examined the relative reliability of amplitude, velocity, and frequency components of sway. Finally, it is important that a new objective test of postural control is validated with clinical scales. For patients with PD, the most common clinical measure of balance impairment is the postural instability and gait disability (PIGD) subscore of the Unified Parkinson’s Disease Rating Scale (UPDRS) [28].

The goal of this study was to develop and validate a practical tool that allows clinicians to measure postural sway in a clinical setting with body-worn accelerometers. We call our tool the instrumented sway system (ISway). Our vision is that this tool will provide reliable, automatic analysis of sway that is sensitive, accurate, robust, and consistent, without the need for clinical experts to deal with the raw data. To achieve this objective, we carried out two studies in order to determine: i) the sensitivity and experimental concurrent validity of ACC compared to force-plate measures of postural sway; and ii) test-retest reliability and clinical concurrent validity of ACC-based measures compared to the PIGD. From this information, we recommend a subset of the most sensitive, reliable, and valid ISway measures to characterize postural control in PD.

## Methods

### Study design

We carried out two studies. The first study was performed in the motion analysis laboratory, to determine the sensitivity and experimental concurrent validity of ACCs compared to force-plate measures (Study I). The second study took place in the hospital neurology clinic to determine the test-retest reliability and clinical concurrent validity of the proposed automatic clinical system, ISway (Study II).

### Subjects

#### Study I: sensitivity and experimental concurrent validity

To assess the sensitivity of sway measures to detect untreated PD and the relationship between ACC and COP measures, 13 subjects with idiopathic PD and 13 age-matched healthy control subjects participated in the study (Table 1). One control subject was excluded because he was diagnosed with a neurological disorder after he had been selected to participate, leaving the control group with 12 subjects. Only subjects with PD who were early- to mid-stage in the disease course and had never been treated with dopaminergic or other anti-parkinsonian medication were invited to participate. The PD and control groups showed no significant difference in age or body mass index (BMI) but the PD group had more male (7) subjects than control group (5).

**Table 1:** Summary of Subject Characteristics for Study I and II

	**n**	**UPDRS**	**Age**	**Medications**
Study I	13 PD	28.1(±11.2)	60.4 ± 8.5 years	None
	12 CTRL		60.2 ± 8.2 years	
Study II	17 PD	28.3 (±10.4)	67.1 ± 7.3 years	PD ON medication
	17 CTRL		67.9 ± 6.1 years	

#### Study II: test-retest reliability and clinical concurrent validity

To assess test-retest reliability of the measures and the relationship with clinical scales, a different group of 17 PD and 17 age-matched control subjects were tested in the neurology clinic by a research assistant. The patients with PD were tested in their ON medication condition (Table 1). The PD and control groups showed no significant difference in age or BMI, but the PD group had more males (12) than the control group (6).

In both studies, subjects were excluded if they presented with any neurological disorders other than PD or if they had any other condition that could affect their balance. Patients were clinically rated by a trained examiner on the Motor Section (III) of the UPDRS and the Hoehn and Yahr Scale immediately before the experimental sessions. The UPDRS Part III consists of 23 items related to bradykinesia, rigidity, tremor and posture and gait signs of PD, rated on a 4-point scale [28]. The PIGD consists of the sum of 4 UPDRS sub-items, posture, gait, sit-to-stand and pull test, with score from 0 (normal) to 16 (severe) [7].

### Measurement protocol and data acquisition

#### Study I: sensitivity and experimental concurrent validity

All participants were instructed to maintain an upright standing position on a force-plate (AMTI OR6-6, Watertown, MA), with arms crossed and heel-to-heel distance fixed at 10 cm. Feet were allowed to be externally rotated at a comfortable amount for each subject [29], and all the participants wore similar athletic shoes. Initial stance position was consistent from trial to trial by tracing foot outlines on the force-plate.

Subjects wore a MTX Xsens sensor (49A33G15, Xsens, Enschede, NL) with 3-D accelerometers (±1.7 g range) mounted on the posterior trunk at the level of L5, near the body center of mass. The sensing axes were oriented along the anatomical antero-posterior (AP), medio-lateral (ML), and vertical directions. A total of three, 2-minute trials were performed with eyes open gazing straight ahead at an art poster.

The COP displacement was calculated from the ground reaction forces recorded by the force-plate at a 100-Hz sampling frequency and after applying a 10-Hz cut-off, zero-phase, low-pass Butterworth filter. Acceleration signals were collected with a 50-Hz sampling frequency, transformed to a true horizontal-vertical Cartesian coordinate system [30] and filtered with a 3.5 Hz cut-off, zero-phase, low-pass Butterworth filter. This filter was applied also to the COP in order to eliminate possible contributions of tremor at rest, a well-known PD symptom, which may be present in the range from 4-to-7 Hz [31].

#### Study II: test-retest reliability and clinical concurrent validity

Subjects wore the same MTX Xsens sensors on the posterior trunk at the level of L5. To ensure a consistent foot-width position, we constructed a styrofoam wedge that was placed between the feet before each trial. Three 30 s trials of quiet standing were collected.

The sensor was removed after finishing the 3 ISway trials. After 30 min resting in a chair the sensor was put back on and the protocol was repeated. We assumed that the subjects’ performances remained the same within this time period. The same examiner used the same device and the same protocol to test the subject for the second time.

### Automatic instrumented Sway in the clinic: ISway

For the experiments in the clinic, subjects wore a portable data-receiver (X-Bus) wired to the MTX XSens sensors. The sensor recorded 3-D linear accelerations and angular velocity while the controller continuously, wirelessly streamed data to a laptop via Bluetooth.

A custom MATLAB (MathWorks, Nantick, MA) graphical interface was built to acquire, store and analyze different components of balance. The software also automatically compared each subject’s balance-related measures compared to normative ranges (based on metrics from healthy subjects) and uploaded the data to a server for additional analysis.

In addition to the previously described pre-processing of the acceleration signals, the algorithm includes an automatic inspection of acceleration signals to make sure that trials in which subjects may have lost their balance were excluded from the analysis. Specifically, signals were divided into three 10s windows and the standard deviation (SD) was computed on each window. If the SD of at least one of the three windows exceeded 5 times the SD of one of the other windows, the trial was discarded. After this automatic check, a total of 13 ACC measures were computed from the 2D acceleration time series, similar to COP analysis (details in Table 2). In the time-domain, we computed six measures that characterized the ACC trajectory and one measure that estimated the area covered by the 2D ACC trace. In the frequency-domain, spectral properties were assessed by five measures: one measure that quantifies the total power of the ACC signal, one measure that estimates the variability of the frequency content of the power spectral density of the ACC, and three measures of characteristic frequencies in the power spectral density of the ACC signal. We included jerkiness of sway, as described in a previous paper [10].

**Table 2:** Summary of the extracted measures

***Measure abbreviation***	***Description***
JERK	Sway jerkiness, time derivative of acceleration [m^2^/s^5^] $JERK=\frac{1}{2}\int_{0}^{t}{\left(\frac{dACCL_{\mathrm{AP}}}{dt}\right)}^{2}+{\left(\frac{dACCL_{\mathrm{ML}}}{dt}\right)}^{2}$
***Time-domain measures***	
DIST	Mean distance from center of COP (ACC) trajectory [mm] ([m/s^2^])
RMS	Root mean square of COP (ACC) time series [mm] ([m/s^2^])
PATH	Sway path, total length of COP (ACC) trajectory [mm] ([m/s^2^])
RANGE	Range of COP displacement (acceleration) [mm] ([m/s^2^])
MV	Mean velocity COP: PATH/trial duration [m/s]; ACC: $\sqrt{{\left(\int ACCL_{\mathrm{AP}}\right)}^{2}+{\left(\int ACCL_{\mathrm{ML}}\right)}^{2}}$ [mm/s]
MF	Mean frequency, the number, per second, of loops that have to be run by the COP (ACC), to cover a total trajectory equal to PATH (MF = PATH/ (2*π*DIST*trial duration) (Hz)
AREA	Sway area, computed as the area spanned from the COP (ACC) per unit of time [mm^2^/s] ([m^2^/s^5^])
***Frequency-domain measures***	
PWR	Total power [mm^2^] ([m^2^/s^4^])
F50	Median frequency, frequency below which the 50% of PWR is present (Hz)
F95	95% power frequency, frequency below which the 95% of PWR is present (Hz)
CF	Centroidal frequency (Hz)
FD	Frequency dispersion (−)

### Data analysis

Algorithms for signal analysis and statistical evaluation of the outcomes were written in MATLAB. Differences between untreated PD and control groups were determined by using a t-test. Differences were assumed significant when P < 0.05. A Pearson Product moment correlation was used to assess both the relationship between COP and ACC metrics in Study I and the relationship between ACC metrics and clinical scores (UPDRS III and sub-items) in Study II. To evaluate test-retest reliability of the ISway, Intra-Class Correlation (ICC) was used [32]. Since the same subjects and same device was used for reliability, an ICC(1,1) was used. The ρ and 95% confidence intervals were reported.

## Results

### Study I: sensitivity and experimental concurrent validity

**Figure 1:**
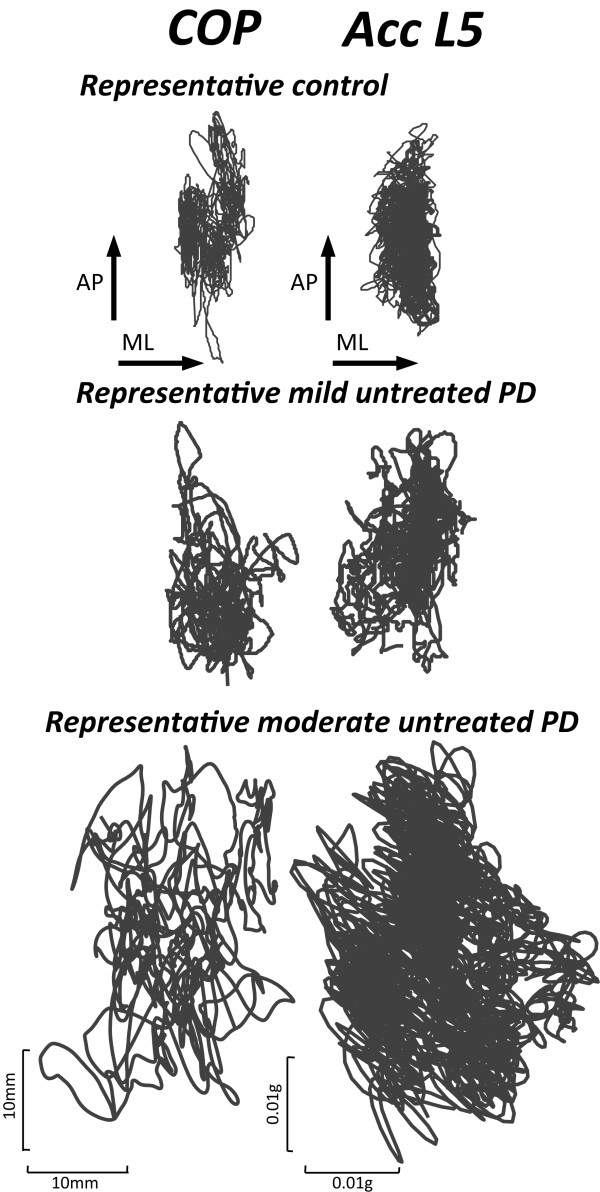
Center of pressure (left panel) and acceleration (right panel) traces in the horizontal plane for three representative subjects.

ACC-based measures of sway were just as sensitive as COP measures in differentiating between the untreated PD and control groups. The COP and ACC traces of a representative control subject, a mild, untreated PD subject (UPDRS = 17), and a moderate, untreated PD subject (UPDRS = 35) during a quiet stance trial are illustrated in Figure 1. The size and jerkiness of both the COP and ACC traces are larger in the mild untreated PD subject and even larger in the moderate, untreated PD subject compared to control subject.

Consistent with the representative trials in Figure 1, most time- and frequency-domain measures of both ACC and COP were sensitive to untreated PD. Table 3 summarizes p-values of the time- and frequency-domain outcome measures comparing untreated PD and control subjects, as well as the correlations between ACC and COP measures.

**Table 3:** Sensitivity of COP and ACC-based measures

	**COP**					**ACC**					**Correlation**	
	**Control**		**PD**		**p values**	**Control**		**PD**		**p values**	**r**	**p**
	**Mean**	**SEM**	**Mean**	**SEM**		**Mean**	**SEM**	**Mean**	**SEM**			
Jerk					NA	0.241	0.023	0.394	0.053	**0.0003**		
**Trajectory Measures**												
DIST	**4.370**	**0.329**	**6.261**	**0.423**	**0.002**	**0.062**	**0.005**	**0.088**	**0.010**	**0.02**	**0.73**	0.0001
RMS	**5.141**	**0.371**	**7.329**	**0.552**	**0.003**	**0.073**	**0.006**	**0.106**	**0.012**	**0.02**	**0.74**	0.0000
PATH	944.015	89.277	817.707	62.661	0.25	17.183	1.028	22.035	2.128	0.05	0.46	0.200
RANGE	32.247	2.750	40.824	3.385	0.06	**0.421**	**0.031**	**0.610**	**0.065**	**0.01**	**0.64**	0.0007
MV	7.867	0.744	6.814	0.522	0.26	1.328	0.229	1.789	0.241	0.18	0.12	0.56
MF	**0.290**	**0.018**	**0.181**	**0.016**	**0.0001**	0.393	0.029	0.378	0.045	0.78	0.33	0.11
**Area Measures**												
AREA	10.699	1.581	13.138	2.121	***0.36***	**0.002**	**0.0003**	**0.005**	**0.001**	**0.004**	**0.68**	0.002
**Frequency Measures**												
PWR	**9870.8**	**1761.7**	**12966.1**	**1585.1**	0.2	**2.009**	**0.269**	**3.178**	**0.479**	**0.04**	0.41	0.05
F50	**0.410**	**0.015**	**0.298**	**0.012**	**0.00009**	**0.385**	**0.014**	**0.305**	**0.012**	**0.0003**	**0.86**	0.000001
F95	**1.518**	**0.069**	**0.990**	**0.057**	**0.0007**	1.849	0.121	1.523	0.128	*0.07*	**0.49**	0.01
CF	**0.666**	**0.032**	**0.451**	**0.023**	**0.0002**	**0.722**	**0.043**	**0.591**	**0.038**	**0.03**	**0.47**	0.02
FD	**0.767**	**0.009**	**0.827**	**0.009**	**0.00005**	**0.777**	**0.009**	**0.839**	**0.008**	**0.0003**	**0.89**	0.000001

Those measures in boldface were significantly different between untreated PD and control subjects. Correlations between COP and ACC measures are also reported.

Most ACC measures of sway were significantly correlated with COP measures of sway, attesting to their concurrent validity (Table 3). Only two measures, MV and MF were not significantly correlated with COP.

### Study II: test-retest reliability and clinical concurrent validity

Table 4 summarizes the test-retest reliability of ACC measures for the control and PD groups. Overall, the PD group showed higher test-retest reliability than the control group. The JERK and time-domain measures showed the best reliability (highlighted in Table 4), whereas the frequency-domain measures had poorer test-retest reliability.

**Table 4:** Test-Retest Reliability of ACC-based measures

	***CTRL***							***PD***						
	**Test I**		**Test II**		**ICC(1-1)**	**95%Cl bounds**		**Test I**		**Test II**		**ICC(1-1)**	**95%Cl bounds**	
	**Mean**	**SEM**	**Mean**	**SEM**	**ρ**	**lower**	**upper**	**Mean**	**SEM**	**Mean**	**SEM**	**ρ**	**lower**	**upper**
Jerk	**0.065**	**0.007**	**0.067**	**0.007**	**0.87**	0.67	0.95	**0.235**	**0.110**	**0.188**	**0.066**	**0.86**	0.66	0.95
***Trajectory Measures***														
DIST	**0.043**	**0.003**	**0.048**	**0.004**	**0.70**	0.34	0.88	**0.089**	**0.020**	**0.074**	**0.014**	**0.84**	0.61	0.94
RMS	**0.052**	**0.003**	**0.057**	**0.005**	**0.71**	0.35	0.89	**0.108**	**0.026**	**0.089**	**0.017**	**0.83**	0.59	0.93
PATH	**4.428**	**0.220**	**4.484**	**0.234**	**0.89**	0.72	0.96	**8.232**	**1.285**	**7.286**	**0.909**	**0.81**	0.56	0.93
RANGE	**0.275**	**0.016**	**0.283**	**0.020**	**0.74**	0.41	0.90	**0.567**	**0.150**	**0.494**	**0.102**	**0.82**	0.58	0.93
MV	0.104	0.012	0.122	0.013	0.68	0.31	0.87	**0.211**	**0.049**	**0.176**	**0.029**	**0.75**	0.44	0.90
MF	0.58	0.03	0.55	0.05	0.60	0.17	0.84	0.54	0.04	0.54	0.04	0.55	0.12	0.81
***Area Measures***														
AREA	**0.0019**	**0.0002**	**0.0021**	**0.0002**	**0.76**	0.45	0.91	**0.0127**	**0.0059**	**0.0078**	**0.0031**	**0.73**	0.40	0.89
***Frequency Measures***														
PWR	0.41	0.04	0.47	0.08	0.61	0.19	0.84	**3.54**	**2.24**	**2.04**	**1.33**	**0.85**	0.64	0.94
F50	0.43	0.03	0.41	0.05	0.30	-0.20	0.68	0.36	0.01	0.37	0.02	0.35	-0.13	0.70
F95	1.95	0.10	1.92	0.10	0.59	0.17	0.83	1.78	0.16	2.00	0.14	0.67	0.31	0.87
CF	0.78	0.05	0.76	0.05	0.61	0.19	0.84	0.69	0.05	0.76	0.06	0.69	0.34	0.87
FD	0.76	0.01	0.79	0.02	0.25	-0.25	0.65	0.78	0.01	0.80	0.01	0.61	0.20	0.84

Table 5 summarizes the clinical concurrent validity. Several ACC measures showed significant correlation with the PIGD sub-score of the UPDRS III. All except one time-domain measure (MF), one frequency-domain measure (PWR), and JERK were significantly and positively correlated with the PIGD sub-score related to clinical postural instability. No significant correlations were found between ACC measures and the total Motor UPDRS.

**Table 5:** Correlation of ACC measures with UPDRS III and PIGD

	***UPDRS III***	***PIGD***
***JERK***	0.29 (p = 0.29)	**0.55 (p = 0.03)**
***Time-domain measures***		
***DIST***	0.29 (p = 0.29)	**0.57 (p = 0.02)**
***RMS***	0.29 (p = 0.28)	**0.57 (p = 0.02)**
***PATH***	0.22 (p = 0.42)	**0.50 (p = 0.05)**
***RANGE***	0.29 (p = 0.30)	**0.56 (p = 0.03)**
***MV***	0.29 (p = 0.28)	**0.63 (p = 0.01)**
***MF***	−0.09 (p = 0.72)	−0.37 (p = 0.17)
***AREA***	0.29 (p = 0.29)	**0.55 (p = 0.03)**
***Frequency-domain measures***		
***PWR***	0.30 (p = 0.27)	**0.54 (p = 0.04)**
***F50***	−0.17 (p = 0.54)	−0.18 (p = 0.49)
***F95***	−0.24 (p = 0.38)	−0.42 (p = 0.12)
***CF***	−0.18 (p = 0.50)	0.34 (p = 0.20)
***FD***	0.23 (p = 0.41)	0.31 (p = 0.26)

## Discussion

ISway is an innovative tool that can allow clinicians to objectively measure posture control during stance easily and quickly in a clinical setting. The approach of using accelerometers on the belt to quantify postural sway was shown to be sensitive, valid, and reliable for patients with PD.

The JERK of sway acceleration was found to be the most sensitive measure to discriminate untreated PD and control subjects [10]. The ISway measures also showed significant differences between the untreated PD and age-matched control groups for 5 out of 7 time-domain and 4 out of 5 frequency-domain ACC measures. Similarly, significant differences between groups were observed using COP measures: 3 out of 7 time-domain and 4 out of 5 frequency-domain COP measures. Subjects with untreated PD showed larger sway amplitude and area, larger sway jerkiness but lower frequencies of sway than control subjects. A more detailed discussion of ACC differences in postural sway between mild PD and control groups is presented in a separate paper [10].

ISway measures of postural sway were validated by force-plate measures of COP displacement. Many, but not all, ISway measures were correlated with the gold-standard laboratory measures of sway from a force-plate. If the body was moving like an inverted pendulum, a correlation close to 1 would be expected between trunk acceleration and COP displacement [33]. In fact, highly correlated measures of COP and center of mass amplitudes have also been reported during quiet stance [34,35]. Moderate to good correlations were found between COP and ACC parameters, except for MV and MF. One explanation can be that we used a different formula to calculate MV for COP and ACC; for ACC, we integrated the acceleration signal to get the velocity and for COP, we differentiated the COP displacement. Another explanation for the difference may be that subjects do not sway strictly as inverted pendulums [36]. In fact, even quiet stance in young, healthy subjects includes some hip strategy and the amount of hip strategy used to control stance posture have been shown to increase with age.

To our knowledge, this is the first paper reporting reliability of postural sway measures, in control or PD subjects, using an accelerometer-based approach. As expected, ICCs were larger in PD subjects because there was larger intra-subject variability in sway among PD subjects than control subjects, without any increase in variability of performance across sessions. In the PD group, 6 ACC parameters out of 13 showed excellent reliability (ICC > .80), 5 good (.60 < ICC < .80), 1 moderate (.40 < ICC < .60), and 1 poor reliability (ICC < .40). In control subjects, 2 out of 13 ACC parameters showed excellent reliability, 8 good, 1 moderate, and 2 parameters showed poor reliability. In general, JERK and time-domain parameters showed better reliability than frequency-domain parameters, and this has been previously reported in COP based measure of postural sway [37,38]. Although it is possible that the poor reliability of sway frequency metrics in people with PD, compared to other spatial-temporal metrics, could be due to fluctuating tremor, we think this unlikely because we filtered the acceleration signals below 3.5 Hz. In addition, healthy control subjects showed poorer reliability of the frequency metrics. A possible explanation for the worse test-retest reliability of frequency-domain parameters might be the variation in a subject’s balance strategy across the testing sessions, or might be attributed to the shorter trial duration of trials in study II.

The ISway measures showed good clinical validity as the measures are related to clinical scores. JERK, 6 out of 7 time-domain measures, and total power significantly correlated significantly with PIGD, a UPDRS III subscore that is used in clinical trials to evaluate postural instability and gait disability based on clinical rating of postural alignment, the pull test of postural stepping response, sit to stand and gait [39]. However, ISway measures were not significantly related with the overall Motor UPDRS III. Since UPDRS motor scale is composed of scores mostly related to tremor, bradykinesia and rigidity, its poor correlation with ISway might suggest that the balance deficiency in early PD is not explained by those symptoms. In fact, our recent meta-analysis of long-term effects of deep brain stimulation surgery in patients with PD show independent decline in PIGD, even when the rest of the UPDRS cardinal signs slowed their decline after surgery [40].

The potential application of the ISway is not to be limited to testing subjects with PD. The ISway provides a large number of measures that automatically, fully characterize body sway in amplitude, smoothness, and frequency; measures that are relevant for testing any individual with balance deficits. In fact, it is likely that a different subset of measures in the ISway might be sensitive to different neurological or musculoskeletal constraints. For example, JERK has been shown to be lower than normal in patients with mild multiple sclerosis [41]. Further studies are needed to determine the best subset of postural sway parameters that can predict future falls or disability during daily activities.

## Conclusion

Previous results [23,24,42-44] suggest that postural sway could be characterized by three relatively independent characteristics: amplitude, velocity and frequency. Our results showed the most sensitive and reliable ISway measures to characterize posture control in PD are: 1) JERK, for its excellent reliability and sensitivity; 2) RMS for its best sensitivity and reliability among the time-domain measures and MV, for the best correlation with clinical score (even if it is not sensitive to untreated PD); and 3) CF as the best compromise from the frequency-domain measures (sensitive, reliable, and correlated with COP CF).

In summary, the present accelerometric-based measurement of sway offers an inexpensive and efficient alternative for quantifying posture control and provides excellent opportunities for on-line sway analyses. The method is reliable and can be used together with clinical balance and mobility tests in various circumstances, particularly in outcome and screening studies.

## Competing interests

ISway is used in Mobility Lab by APDM. Drs. Horak and Salarian have significant financial interests in APDM, a company that has a commercial interest in the results of this research and technology. This potential conflict of interest has been reviewed and managed by OHSU and the Integrity Oversight Council.

## Authors´ contribution

MM participated to study design and data collection, was responsible for data analysis and interpretation, drafting the manuscript. AS participated to study design and conception, participated in data analysis and interpretation, revising manuscript. PCK, CZ, and LK were responsible for data collection and interpretation, revising critically the manuscript. LC and FBH were responsible for study design and conception, drafting and revising critically the manuscript. All authors read and approved the final manuscript.
